# A Repurposed Drug Screen for Compounds Regulating Aquaporin 5 Stability in Lung Epithelial Cells

**DOI:** 10.3389/fphar.2022.828643

**Published:** 2022-01-25

**Authors:** John Villandre, Virginia White, Travis B. Lear, Yanwen Chen, Ferhan Tuncer, Emily Vaiz, Beyza Tuncer, Karina Lockwood, Dan Camarco, Yuan Liu, Bill B. Chen, John Evankovich

**Affiliations:** ^1^ Department of Medicine, Acute Lung Injury Center of Excellence, University of Pittsburgh, Pittsburgh, PA, United States; ^2^ Aging Institute, University of Pittsburgh, Pittsburgh, PA, United States; ^3^ Vascular Medicine Institute, University of Pittsburgh, Pittsburgh, PA, United States; ^4^ McGowan Institute for Regenerative Medicine, University of Pittsburgh, Pittsburgh, PA, United States

**Keywords:** repurposed drugs, proteasome, Aquaporin 5, Niclosamide, lung epithelial cells

## Abstract

Aquaporin 5 (AQP5) is expressed in several cell types in the lung and regulates water transport, which contributes to barrier function during injury and the composition of glandular secretions. Reduced AQP5 expression is associated with barrier dysfunction during acute lung injury, and strategies to enhance its expression are associated with favorable phenotypes. Thus, pharmacologically enhancing AQP5 expression could be beneficial. Here, we optimized a high-throughput assay designed to detect AQP5 abundance using a cell line stably expressing bioluminescent-tagged AQP5. We then screened a library of 1153 compounds composed of FDA-approved drugs for their effects on AQP5 abundance. We show compounds Niclosamide, Panobinostat, and Candesartan Celexitil increased AQP5 abundance, and show that Niclosamide has favorable cellular toxicity profiles. We determine that AQP5 levels are regulated in part by ubiquitination and proteasomal degradation in lung epithelial cells, and mechanistically Niclosamide increases AQP5 levels by reducing AQP5 ubiquitination and proteasomal degradation. Functionally, Niclosamide stabilized AQP5 levels in response to hypotonic stress, a stimulus known to reduce AQP5 levels. In complementary assays, Niclosamide increased endogenous AQP5 in both A549 cells and in primary, polarized human bronchial epithelial cells compared to control-treated cells. Further, we measured rapid cell volume changes in A549 cells in response to osmotic stress, an effect controlled by aquaporin channels. Niclosamide-treated A549 cell volume changes occurred more rapidly compared to control-treated cells, suggesting that increased Niclosamide-mediated increases in AQP5 expression affects functional water transport. Taken together, we describe a strategy to identify repurposed compounds for their effect on AQP5 protein abundance. We validated the effects of Niclosamide on endogenous AQP5 levels and in regulating cell-volume changes in response to tonicity changes. Our findings highlight a unique approach to screen for drug effects on protein abundance, and our workflow can be applied broadly to study compound effects on protein abundance in lung epithelial cells.

## Introduction

The family of aquaporin proteins (AQP) display tissue and cell-specific expression patterns to regulate water transport across various membranes. In the lung, Aquaporin-1 (AQP1), Aquaporin-4 (AQP4), and Aquaporin-5 (AQP5) are expressed in several cells types (Verkman, 2007; Yadav et al., 2020). One member of this family, Aquaporin-5, is expressed in various epithelial cells in the lung, including AT-1 cells and the bronchial epithelium (King et al., 2000; Wittekindt and Dietl, 2019). AQP5 is implicated in the development of acute lung injury, and genetic polymorphisms in the AQP5 promoter have been associated with clinical outcomes in critically ill humans (Rump et al., 2016). Preclinical models of murine acute lung injury show a pattern where AQP5 protein abundance is reduced in the lung following injury (Towne et al., 2000; Hasan et al., 2014; Vassiliou et al., 2017; Zhang J. et al., 2018), and some studies suggest restoring its expression could be protective (Jiang et al., 2015). AQP5 deficiency has also been implicated in preclinical models of xerostomia, sialadenitis, and Sjogren’s syndrome, and restoration of aquaporin channels in these tissues is associated with increased saliva secretion (Lai et al., 2016). Thus, preventing loss of AQP5 could be beneficial in several pathophysiological models. Given the rationale that increasing AQP5 levels could be beneficial in a number of disease states, we set out to determine whether compounds from a repurposed drug library could affect total AQP5 levels.

AQP5 abundance is dynamically regulated. Several stimuli in the lung affect AQP5 gene transcription, ultimately affecting protein abundance and function. Additionally, changes in AQP5 abundance can also occur at the post-translational level. For example, several other members of the aquaporin protein family have been shown to undergo regulation via degradation in the proteasome. AQP2 undergoes ubiquitination and proteasomal degradation (Lee and Kwon, 2009), as does AQP1 (Leitch et al., 2001). However, regulation of AQP5 abundance by proteasomal degradation has not been described. We hypothesized that similar to other aquaporins, AQP5 undergoes proteasomal degradation. Thus, compounds that interfere with AQP5 degradation could ultimately influence total AQP5 abundance and function. Here, we utilized a screening platform that measured bioluminescent-tagged AQP5 (AQP5-HiBiT) levels as a read-out and identify compounds that influence AQP5 abundance. Mechnistically, we identify that the compound Niclosamide increases AQP5 abundance by reducing AQP5 ubiquitination and disposal in the proteasome.

Drug repurposing is an approach to study approved compounds for their effects outside their initial scope or indication (Pushpakom et al., 2019). Several re-purposed compound libraries are commercially available to screen for their effects on a given biological process, assay, or disease process. These libraries are particularly useful in medium to high-throughput assays, where the effects of a library of compounds can be tested on a very specific biological process. The attractiveness of using a repurposed compound library is such that extensive pharmacokinetic and safety data already exist for all of the compounds in the library, and thus whether a compound is suitable for further clinical development is more easily assessed. We have developed a workflow that directly measures abundance of a given protein, and thus we can screen for the effect of a library of compounds on abundance of a target protein. As there is increasing interest in aquaporins as druggable targets, including Aquaporin-5 (Yadav et al., 2020), we sought out to test the effects of repurposed FDA-approved compounds on AQP5 abundance.

We utilized a high-throughput screening platform that directly measures protein abundance (Chen et al., 2020a). Our design used the split NanoLuc Luciferase system (Promega), which incorporates a small 11 amino acid endogenous tag (SmBiT) onto a protein of interest, and when combined with a detection reagent containing the complementary LgBiT combines for form functional NanoLuc Luciferase. Thus, luminescence is a readout for abundance of HiBiT-tagged protein. In this study, we generated a stable cell line expressing AQP5-HiBiT and optimized an assay to measure AQP5-HiBiT abundance. Since the construct is driven by a CMV promoter rather than the endogenous AQP5 promoter, compounds that affect AP5-HiBiT are likely to affect AQP5 levels independent of AQP5 transcriptional activity. Further, this approach is frequently used to identify compounds that affect protein degradation (Chen et al., 2020a; Simard et al., 2021).

 After assay optimization for high-throughput applications, we screened for compounds that increased AQP5 abundance from a library of 1153 FDA-approved compounds. We focused on three compounds which increased AQP5-HiBiT abundance: Niclosamide, a pleotropic compound approved as an anti-helminthic, Panobinostat, an inhibitor of histone deacetylase activity, and Candesartan, an angiotensinogen receptor blocker. We demonstrate Niclosamide stabilizes AQP5 abundance by preventing its ubiquitination and degradation. *In vitro*, treatment with Niclosamide increased endogenous AQP5 in A549 cells and primary human bronchial epithelial cells cultured on an air-surface liquid interface. Functionally, Niclosamide increased cellular volume changes in response to hypertonic media, an effect dependent on water transporters. The results from these studies can be tested broadly in pathological conditions to modulate AQP5 abundance.

## Materials and Methods

### Cell Culture

Beas-2B cells from ATCC were cultured in HITES media supplemented with 10% fetal bovine serum (FBS) from Gibco. A549 cells from ATCC were cultured in DMEM high glucose, glutamax media supplemented with 10% fetal bovine serum (FBS) from Gibco. For primary bronchial epithelial cell experiments, cells were obtained from the University of Pittsburgh Primary Airway Cell and Assay Core. Following attaining informed consent, airway segments and lung tissue were obtained from excess pathological tissue following lung transplantation in accordance with a protocol approved by the University of Pittsburgh Investigational Review Board.

### HiBiT Subcloning

HiBiT-tagged human AQP5 plasmid constructs were created using the FLEXI system (Promega). The ORF of hAQP5 was amplified via PCR and cloned into the pFN38K HiBiT CMV-neo Flexi® Vector.

### Transfection and Stable Cell Line Generation

The HiBiT AQP5 plasmid was nucleofected into Beas-2B cells using the Nucleofector II (Amaxa) and placed under antibiotic selection. Cells were then seeded into a 384 well plate at an approximate density of 1.5 cells per well. After colony expansion, each well was tested for HiBiT response to CHX and MG-132 treatment. The top 10 colonies were further expanded and validated. The lead colony was expanded and seeded into 384 well plates at an approximate density of 5,000 per well, and treated with CHX and MG-132 to calculate Z′-factor (Zhang et al., 1999).

### High Throughput Liquid Handling

The Agilent Bravo automated liquid-handling platform was used to transfer contents of compound library onto assay plates. A Biotek EL406 washer dispenser was used to distribute reagents or cell solutions into assay plates.

### HiBiT FDA Approved Drug Library Screening

AQP5-HiBiT cells were seeded to a final density of 5,000 cells per well. The FDA-approved compound library (100 nL per drug) was stamped to 384-well tissue culture plates using CyBio Well vario (Analytik Jena). Compounds were plated to the final concentrations of 10 μM. After 18 h of treatment, culture media was removed and cells were processed for Nanoluciferase activity using Nano-Glo^®^ HiBiT Lytic Detection Reagent (Promega, 2021), according to manufacturer’s protocol. Signals were detected and quantified using a ClarioStar Plus plate reader.

### Immunoblotting

Cells were lysed with RIPA buffer including EDTA-free protease inhibitor on ice. Lysates were then sonicated at 20% amplification for 12 s and then centrifuged at 12,000 xg for 10 min at 4 C. Supernatants were collected and normalized for protein concentration via Lowry assay. Samples were then subjected to SDS-PAGE.

### AQP5 HiBiT Detection

AQP5 HiBiT cells were plated at a density of 5,000 cells per well in white 384 well plates. After treatment, cell media was removed and 20 uL of Nano-Glo^®^ HiBiT Lytic Detection Reagent (Promega) was added to each well. Plates were shaken for 10 min, followed by 5 min on the bench to complete cell lyses before reading well luminescence. For indicated experiments, AQP5-HiBiT signal was normalized to cell viability by using ATP-Glo™ Bioluminometric Cell Viability Assay Reagent (Promega). This reagent was added immediately following HiBiT measurements.

### HiBiT blotting

Samples from cells with HiBiT-tagged proteins were prepared following the same protocol as immunoblotting. Proteins were transferred to a nitrocellulose membrane, followed by gentle rocking in TBST to rinse away transfer buffer. Nano-Glo HiBiT blotting system (Promega) was used for development, following manufacturer’s protocol. Briefly, the blot was incubated in 5 ml 1 × Nano-Glo blotting buffer supplemented with 25 μL LgBiT protein overnight at 4 °C. The next day, 10 μL Nano-Glo luciferase assay substrate was directly added into the solution and mixed well immediately. After incubation for 5 min at room temperature in dark, the blot was imaged by ChemiDoc Imaging System (Bio-Rad), using chemiluminescence mode.

### Reagents

AQP5 (HPA065008) antibody, sucrose, and Niclosamide were obtained from Millipore-Sigma. β-Actin mouse monoclonal antibody (sc-81178) was obtained from Santa Cruz Biotechnologies. CHX was obtained from Calbiochem. Carfilzomib (17554) was from Cayman Biochemical. Panobinostat (S1030) and Candesartan (S1578) were obtained from Selleck Chemicals LLC. MG132 (F1100) was obtained from UBPBio. Calcein, AM (C1430) was obtained from Thermo Fisher.

### TUBEs Pulldown

Cells were lysed with IP buffer (0.25% Triton X-100 in 1 × PBS supplemented with EDTA-free protease inhibitor tablet) on ice. Lysates were then sonicated at 20% amplification for 12 s and centrifuged at 12,000 g for 10 min under 4°C. Supernatants were collected and 10% of the volume was saved as input. Tandem-ubiquitin binding complexes were then captured with 50 μL TUBEs per 700 μL lysate for 2 h. After washing with 1 ml TBS-T buffer twice, protein was eluted in 50 mM Tris HCl pH 6.8, 2% SDS, 10% glycerol, and 100 mM DTT. After 5 min incubation at 95°C, samples were collected and used for subsequent immunoblotting.

### Calcein Cell Volume Assay

Cell volume changes were assayed via the calcein fluorescence assay, modified from published protocols (Hamann et al., 2002; Mola et al., 2009). Briefly, A549 cells were plated in black Corning 96 well glass bottom plates at a density of 20,000 cells per well. The following day, cells were treated with vehicle control or Niclosamide (2 μM) for 6 h. Cells were loaded with Calcein-AM, which was added to each well to a concentration of 5 μM for 1 h. Media was removed and replaced with 300 mOsm PBS. The plate was transferred into a ClarioStar Plus plate reader. Using the injector function, solutions of various tonicities were added to wells. For experiments testing the effect of Niclosamide, wells were brought to a final osmolarity of 400 mOsm; in the gradient experiment, wells were brought to a final osmolarity of either 400, 420, or 450 mOsm.

### Statistics

All data were analyzed in Prism Graphpad V 8.0. Dose-response curves were generated using [agonist] *vs*. response–variable slope (four parameters) Least Squares Fit Function. AQP5-HiBiT abundance comparisons were analyzed by one-way ANOVA with correction for multiple comparisons, or by Student’s t-test, as indicated.

## Results

### Development of an Assay to Measure AQP5 Abundance

**FIGURE 1:**
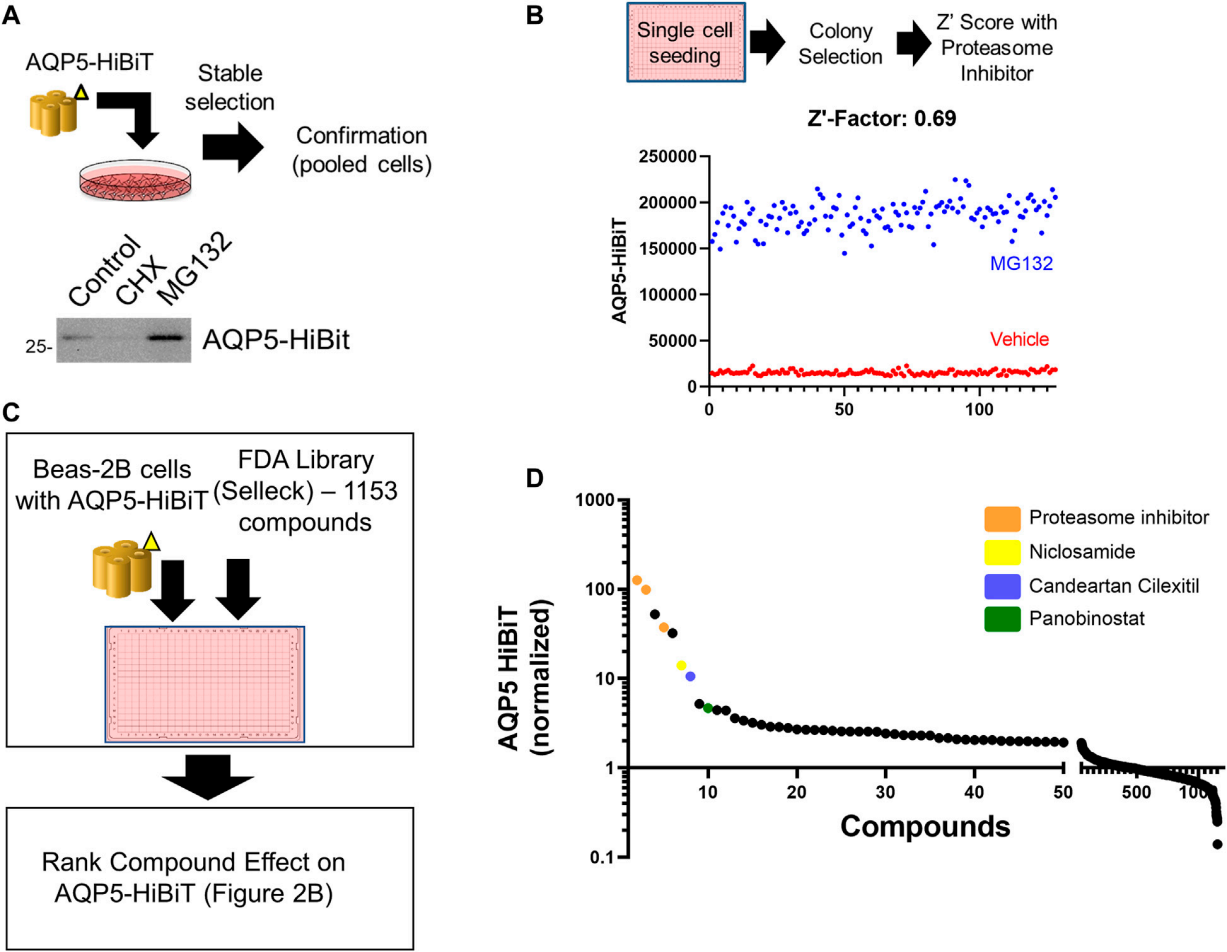
Development of an assay to measure AQP5 Abundance. **(A)** Overview of AQP5-HiBiT stable cell line generation. HiBiT blotting shows AQP5-HiBit construct response to cycloheximide (CHX) and MG-132 in pooled cells. **(B)** AQP5-HiBiT signal in cells were treated with vehicle or MG-132 (*n* = 128 wells/condition) used for Z′ score calculation. **(C)** Overview of screening procedure. AQP5-HiBit cells were plated in 384-well plates and treated with 1153 compounds from the Selleck FDA drug library. AQP5-HiBiT luminescence values were recorded. **(D)** Drug screen results sorted by ranked AQP5-HibiT luminescence, with compounds of interest colorized as indicated.

Beas-2B cells are an immortalized human bronchial epithelial cell line utilized as a model for several pulmonary diseases (Sha et al., 2004), and we have utilized Beas-2B cells for other high-throughput screening applications (Chen et al., 2020a). Full length AQP5 (Sequence Accession BC032946) was sub-cloned into Promega vector pFN38K HiBiT CMV-neo Flexi^®^ Vector (Promega, 2021). AQP5-HiBiT-transfected Beas-2B cells were placed under antibiotic selection to create stably expressing AQP5-HiBiT cells. After transfection and antibiotic selection, AQP5-HiBiT expression was first confirmed in pooled cells treated with either the global protein translation inhibitor cycloheximide (CHX) or the proteasome inhibitor MG132. Cell lysates were separated by electrophoresis and assayed for HiBiT (nanoluciferase) signal on a nitrocellulose membrane. There was a corresponding nanoluciferase signal at 28 kDa, the molecular weight of AQP5. Further, the HiBiT signal was blunted in cells treated with CHX and increased in cells treated with the proteasome inhibitor MG132 (Figure 1A), indicating the stably transfected constructs were subject to the effects of translation inhibition with CHX and proteasome inhibition with MG132. Cells were subsequently plated in 384 well plates in single cell suspensions. Clonal populations were developed in the plate and over 10 colonies were tested to select cells that stably expressed AQP5-HiBiT. A Z′ score was calculated after treatment with MG132 for each colony, and we selected a monoclonal cell line (AQP5-HiBiT) for subsequent experiments. The Z’ score for vehicle vs. MG132 in these cells was 0.69, indicating excellent assay properties (Zhang et al., 1999) for high-throughput screening (Figure 1B).

### Several Repurposed Compounds Increase AQP5 Abundance

Next, we sought to determine repurposed compounds that would increase AQP5-HiBiT. We used a repurposed drug library of 1153 FDA-approved compounds (Selleck). Cells were treated with 10 µM compound for 16 h, and AQP5-HiBiT levels assayed via luciferase. We ranked compounds by their effect on AQP5-HiBiT signal (Figure 1C). The top hits were proteasome inhibitors (carfilzomib and bortezomib), as well as the compound MLN2238, which acts to inhibit ubiquitination of proteins and prevent their degradation in the proteasome. Thus, compounds that blocked proteasomal degradation (carfilzomib and bortezomib) and a compound that blocks ubiquitination of substrates (MLN2238) were most effective in increasing AQP5-HiBiT. Treatment of cells with these agents would be expected to increase abundance of proteins undergoing basal proteasomal-mediated degradation, and their effects are not specific to AQP5. However, there were also several other agents that increased AQP5 HiBiT (Figure 1D; Table 1). Complete results from the screen are contained in the data supplement.

**TABLE 1:** Compounds that increased AQP5-HiBiT. Units are expressed as the ratio of compound-treated AQP5-HiBiT (nano-luc) signal/vehicle-treated (nano-luc) signal. Selected clinically relevant compounds selected for validation are bolded.

Compound	AQP5-HiBiT (nano-luc)/Vehicle (nano-luc)	Compound	AQP5-HiBiT (nano-luc)/Vehicle (nano-luc)
Carfilzomib (PR-171)	126.11	Mometasone furoate	3.60
Bortezomib (PS-341)	98.69	Dendrid	3.38
Zinc pyrithione	52.44	Vincristine sulfate	3.21
MLN2238(Ixazomib)	37.26	Piperacetazine	3.04
Arsenic oxide	32.27	Digoxin	2.89
**Niclosamide**	**14.07**	Podophyllotoxin	2.88
**Candesartan Cilexetil**	**10.61**	Nilotinib (AMN-107)	2.81
Crystal Violet	5.21	Vinblastine sulfate	2.71
**Panobinostat (LBH589)**	**4.67**	Mitoxantrone hydrochloride	2.68
Gramicidin	4.45	Albendazole	2.66
Bismuth subsalicylate	4.39	Colchicine	2.65

**FIGURE 2:**
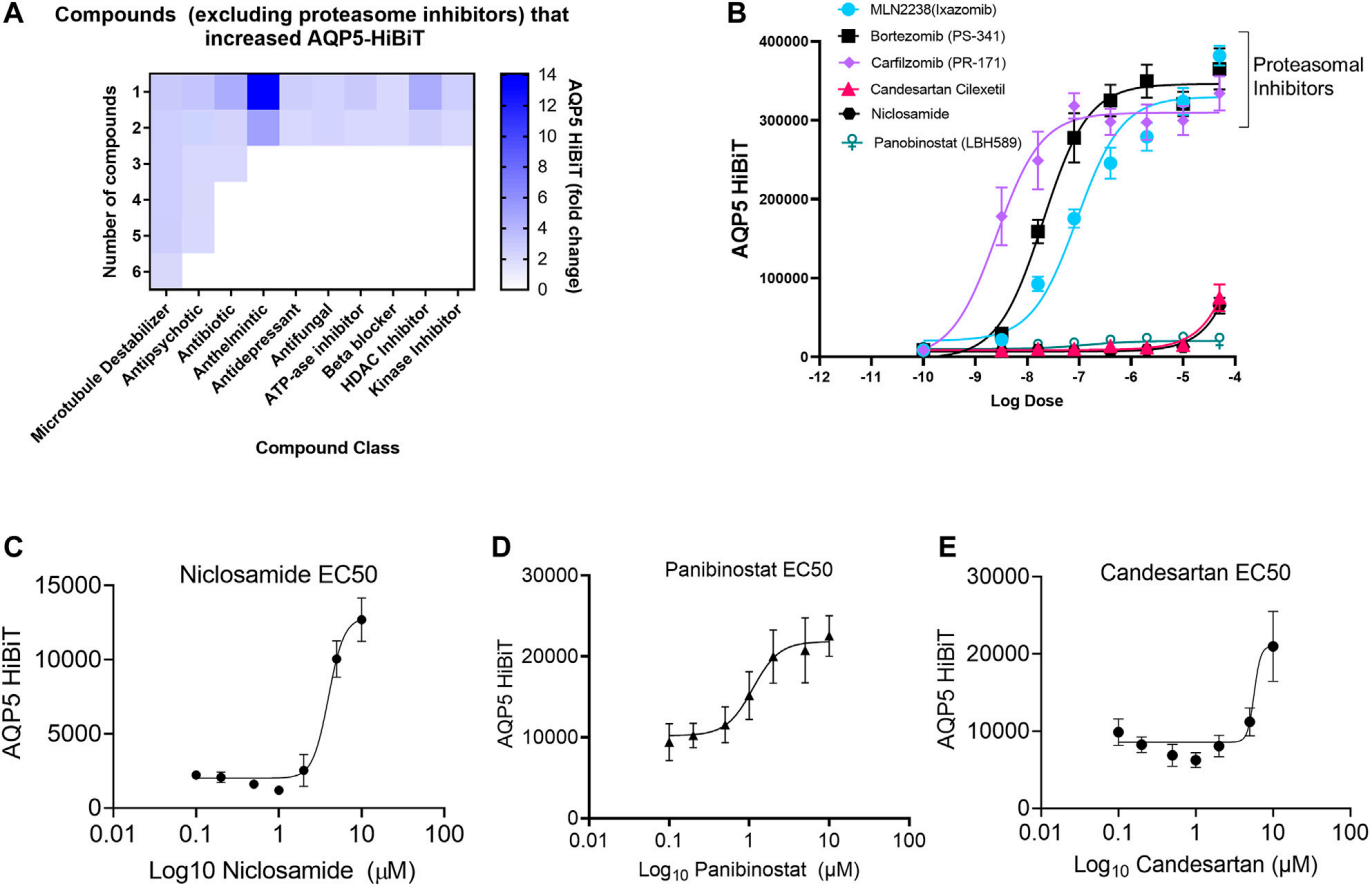
Niclosamide, candesartan, and Panobinostat show dose-dependent accumulation of AQP5-HiBiT. **(A)** Heatmap showing breakdown of compound response classified by drug class. **(B)** Dose response curves of selected proteasome inhibitors (bortezomib, carfilzomib) and E1 inhibitor MLN2238, along with top-hit compounds candesartan, Niclosamide, and Panobinostat. **(C,D, and E)** Dose response curves for Niclosamide, Panobinostat, and candesartan in AQP5-HiBiT (EC50 values of 3.967, 1.109, and 5.687 μM respectively).

Drug classes that increased AQP5 were broad, and included compounds classified by several different primary indications (Figure 2A). To validate findings from the screen, dose response curves were generated for the top compounds, including those that interfered with proteasomal-mediated protein degradation (MLN2238, bortezomib, and carfilzomib) and the top three clinically relevant compounds that increased AQP5 abundance (Niclosamide, Panobinostat, and Candesartan). The non-specific protein-degradation compounds MLN2238, bortezomib, and carfilzomib dramatically increased AQP5 at low doses (Figure 2B). The effects of Niclosamide, Panobinostat, and Candesartan were less pronounced, and we went on to create additional dose-response curves for the three compounds at different doses (Figures 2C–E).

### Cellular Toxicity of Top Hit Compounds

**FIGURE 3:**
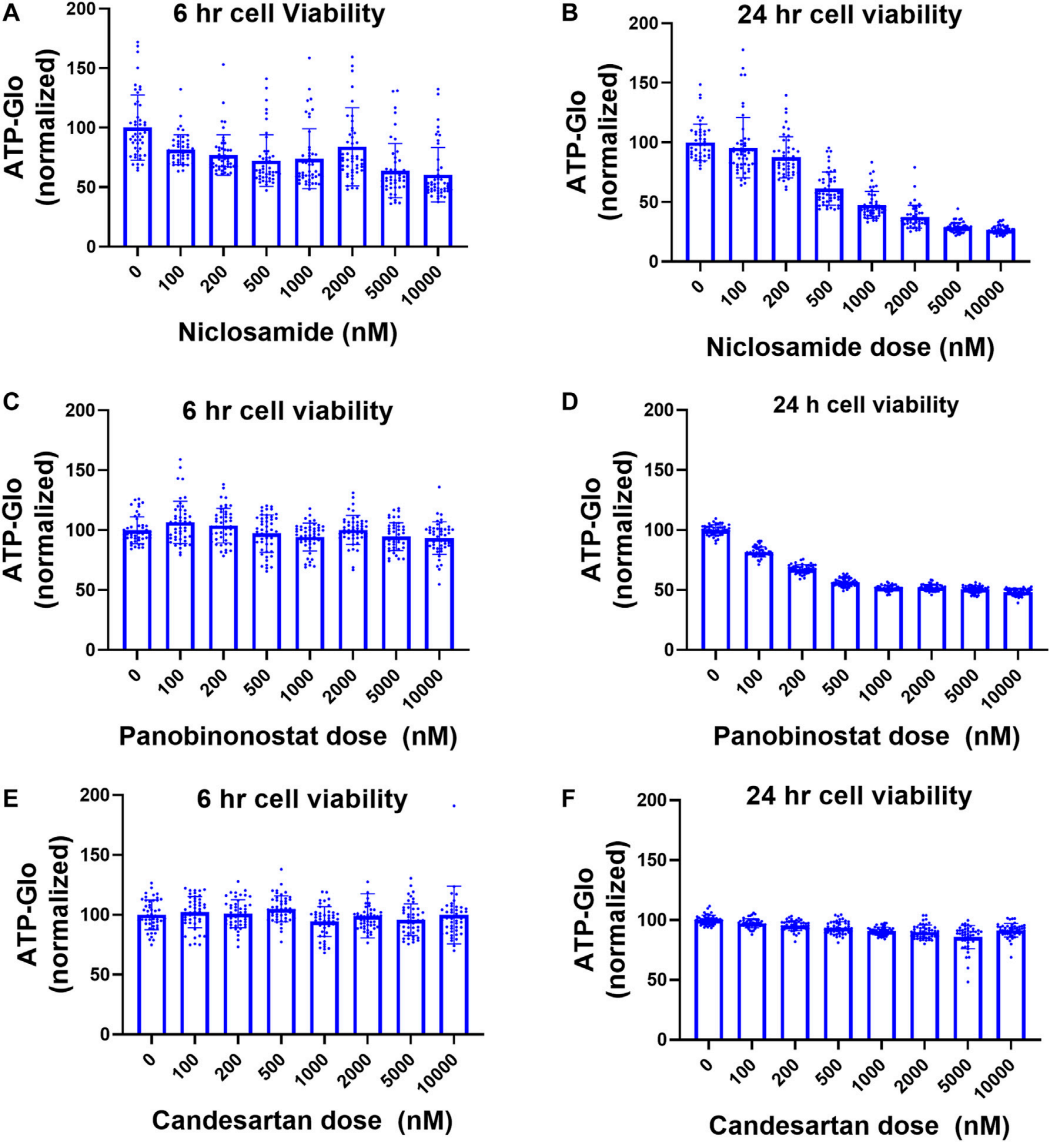
Cell viability after treatment with Niclosamide, candesartan, and Panobinostat. **(A,B)** Viability of AQP5-HiBiT cells in response to 6 h **(A)** and 24 h **(B)** Niclosamide at various doses. **(C,D)**. Viability of AQP5-HiBiT cells in response to 6 h **(C)** and 24 h **(D)** Panobinostat at various doses. **(E,F)** Viability of AQP5-HiBiT cells in response to 6 h **(E)** and 24 h **(F)** candesartan at various doses.

We evaluated the toxicity of Niclosamide, Panobinostat, and Candesartan in our system using ATP-Glo at 6 and 24 h after drug treatment. Niclosamide had little appreciable effects on cell viability at doses less than 2 μM after 6 h, but a dose of 1 μM caused >50% cell death after 24 h (Figures 3A,B). Panobinostat did not affect cell viability at any tested doses after 6 h, but at 24h reduced viability by 50% at a dose of 500 nM (Figures 3C,D). Candesartan had no significant effect on cellular viability at 6 h or 24 h (Figures 3E,F).

### Niclosamide Stabilizes AQP5

**FIGURE 4:**
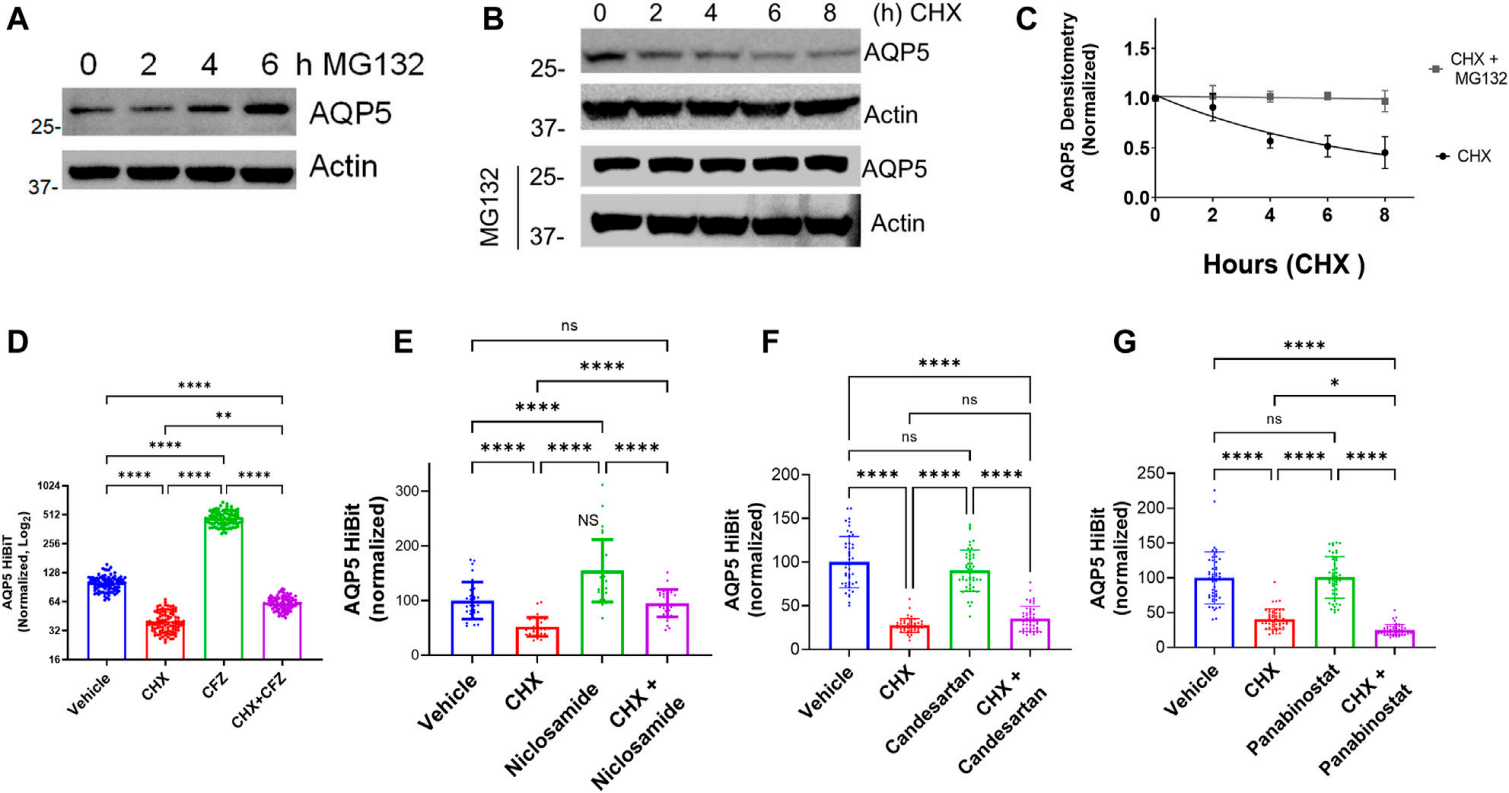
AQP5 is degraded in the proteasome. **(A)** Immunoblot analysis of accumulation of AQP5 in Beas-2B cells in response to a MG-132 at various time points. **(B)** Time course immunoblot analysis of AQP5 with or without MG-132 co-treatment. **(C)** Decay curve of AQP5 abundance in Beas-2B treated with cycloheximide, with or without cotreatment with MG-132. Densitometry data are mean ± SEM of 3 independent experiments. **(D)** Normalized AQP5-HiBiT luminescence in response to 6-h treatment with CHX, carfilzomib (CFZ), or cotreatment. N = 96 wells/condition. Graph is representative of repeated independent experiments. **(E)** Normalized AQP5-HiBiT luminescence in response to 6-h treatment with CHX, Niclosamide, or cotreatment. N = 32 wells/condition. Graph is representative of repeated independent experiments. **(F)** Normalized AQP5-HiBiT luminescence in response to 6-h treatment with CHX, candesartan, or cotreatment. N = 48 wells/condition. Graph is representative repeated independent experiments. **(G)** Normalized AQP5-HiBiT luminescence in response to 6-h treatment with CHX, Panobinostat, or cotreatment. N = 48 wells/condition. Graph is representative of repeated independent experiments. **(D–G)**. *p <0.05, **p <0.01, *** *p* <.001, ****p <0.0001 by 1-way ANOVA with correction for multiple comparisons by Tukey’s method.

Our initial screening strategy was developed using cell lines stably expressing a CMV promoter-driven AQP5 construct with the read-out being protein abundance. Top hits from the drug screen were agents that inhibited protein degradation through the proteasome, with non-specific effects on AQP5. We have observed similar results using this strategy for other protein targets (Chen et al., 2020a; Chen et al., 2020b). Thus, we hypothesized that the observed effects of Niclosamide on AQP5 abundance might be mediated through alteration of AQP5 proteasomal degradation. AQP5 degradation in lysosomes in response to hypotonic cell culture media has been reported (Sidhaye et al., 2006), but whether AQP5 undergoes proteasomal degradation has not yet been examined. Notably, other Aquaporin family members, including AQP2 (Kamsteeg et al., 2006) and AQP1 (Leitch et al., 2001) are processed in the proteasome. AQP5 accumulated when Beas-2B cells were treated with the proteasome inhibitor MG132 (Figure 4A). In cycloheximide chase experiments, AQP5 half-life was approximately 6 h, and MG132 stabilized AQP5 levels in the presence of cycloheximide (Figures 4B,C). We next examined if the compounds identified from our screen would preserve AQP5-HiBiT levels after 6 h of treatment, the observed half-life of endogenous AQP5. In AQP5-HiBiT Beas-2B cells, CHX reduced AQP5-HiBiT, while the proteasome inhibitor carfilzomib (CFZ) increased AQP5-HiBiT. Importantly, co-treatment of CHX and CFZ blunted the effect of CHX on AQP5-HiBiT (Figure 4D). These data suggest the predominant mode of AQP5-HiBiT degradation under these conditions is through the proteasome. We tested whether Niclosamide, Panobinostat, or Candesartan could similarly prevent the reduction in AQP5-HiBiT abundance mediated by CHX treatment. Indeed, while Niclosamide treatment increased AQP5-HiBiT after 6 h, co-treatment with CHX and Niclosamide prevented CHX-mediated reduction in AQP5-HiBit abundance (Figure 4E). In contrast, co-treatment with either Panobinostat or Candesartan did not preserve AQP5-HiBiT levels in the presence of CHX (Figures 4F,G). Importantly, Niclosamide has also been shown to have effects on endosomal/lyosomal function (Jurgeit et al., 2012), and AQP5 can be processed in lysosomes (Sidhaye et al., 2006). In complementary experiments, we also tested whether the lysosomal inhibitor leupeptin could increase AQP5-HiBiT levels. In contrast the dramatic effect of the proteasomal inhibitor CFZ (Figure 4D), leupeptin had only a modest effect on AQP5 HiBiT levels, and the effect of Niclosamide was greater (Supplementary Figure S1A). Additionally, modulators of lysosomal function (chloroquine, hydroxychloroquine) did not have significant effects on AQP5 abundance in our initial screen. Thus, we observed that Niclosamide could stabilize AQP5-HiBiT, and we chose to validate the effects of Niclosamide in further experiments.

### Niclosamide Reduces AQP5 Ubiquitination and Increases AQP5 in vitro

**FIGURE 5:**
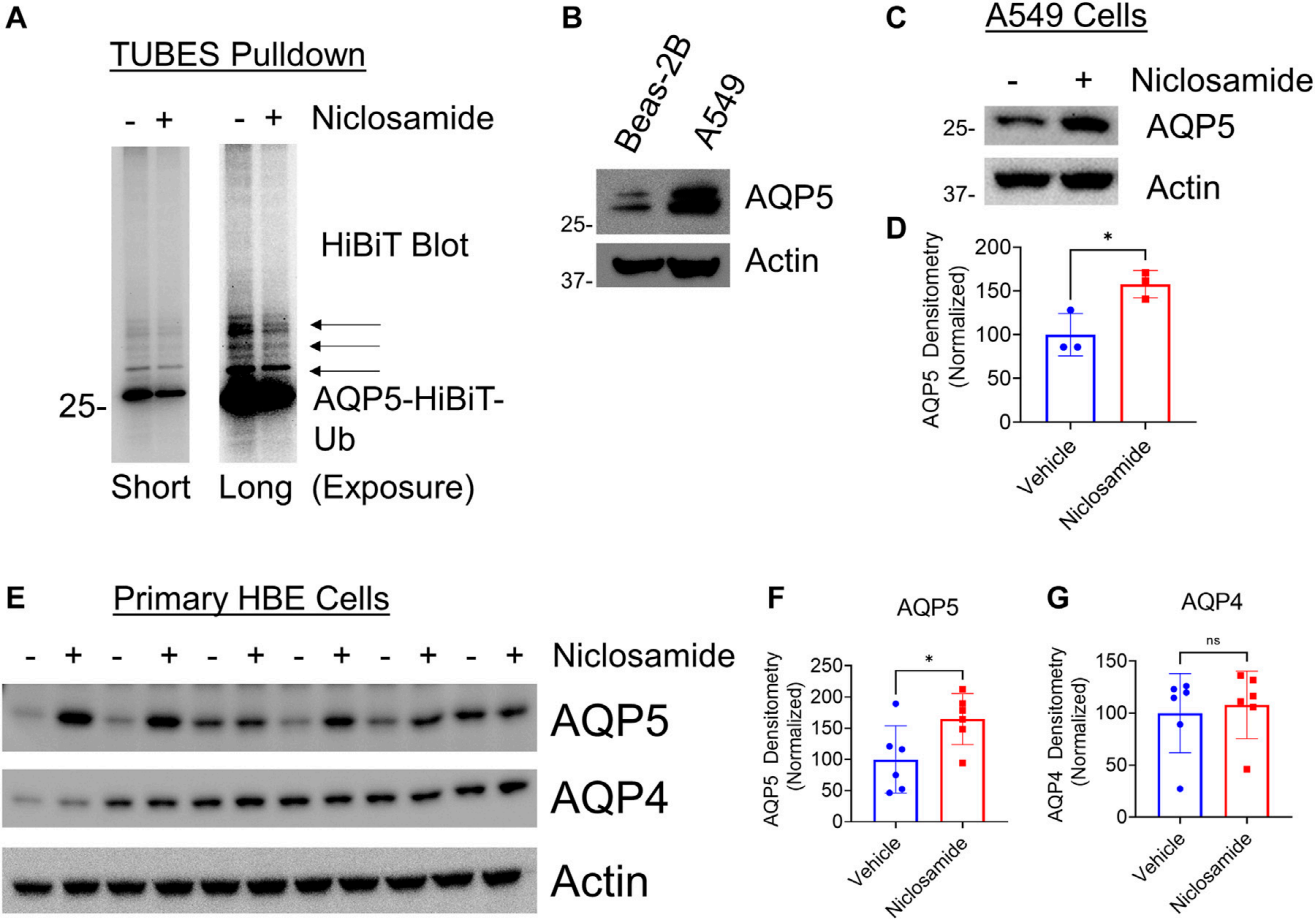
Niclosamide reduces AQP5 ubiquitination and increases endogenous AQP5 levels in A549 cells and primary HBE cells: **(A)** AQP5-HiBiT cells were treated with CFZ and either) vehicle or Niclosamide (200 nM for 16 h, followed by TUBES pulldown to capture ubiquitinated substrates. Pull-down fractions were separated by electrophoresis and HiBiT luminescence was captured on the nitrocellulose membrane. Short exposure (left) shows a diminished primary band at ∼30 kDa in Niclosamide-treated cells, representing mono-ubiquitinated AQP5. A long exposure (right) shows diminished higher molecular weight bands (arrows) in Niclosamide-treated cells, representing multi- and poly-ubiquitinated AQP5. **(B)** AQP5 immunoblot in Beas-2B and A549 cells. **(C)**. AQP5 immunoblot in Vehicle or Niclosamide (2 μM)-treated A549 cells at 6 h. **(D)** Densitometry of AQP5 in vehicle vs. Niclosamide treated cells from n = 3 independent experiments. * *p* = 0.03 by unpaired *t*-test. **(E)** AQP5 or AQP4 immunoblot in Vehicle or Niclosamide (200 nM)-treated primary human bronchial epithelial cells at 16 h. **(F,G)** Densitometry of AQP5 or AQP4 in vehicle *vs*. Niclosamide-treated cells from *n* = 6 wells/condition. * *p* = 0.04, ns = not significant by unpaired *t*-test.

We hypothesized that Niclosamide would have an effect on AQP5 ubiquitination, as ubiquitination is the primary post-translational modification that regulates protein degradation in the proteasome (Komander and Rape, 2012). Using AQP5-HiBiT cells, we treated with the proteasome inhibitor carfilzomib (CFZ) to allow for accumulation of ubiquitinated substrates, with or without co-treatment with Niclosamide. We enriched for ubiquitinated proteins using TUBES pull-down, and after electrophoresis imaged the membrane for HiBiT (nano-luciferase) signal. After TUBES pull-down the nano-luciferase signal is specific for ubiquitinated AQP5, and treatment with Niclosamide reduced AQP5 ubiquitination (Figure 5A), suggesting that its effect on AQP5 abundance may be mediated by preventing its ubiquitination and degradation in the proteasome. Notably, Niclosamide had no effect on total cellular ubiquitin levels (Supplementary Figure S1B).

Next, we sought to determine if Niclosamide would affect abundance of endogenous AQP5. We confirmed that Niclosamide did not affect AQP5 transcript levels (Supplementary Figure S1C). Next, compared to Beas-2B cells, A549 cells had relatively higher baseline expression of AQP5 as determined by immunoblot (Figure 5B), and A549 cells have been used to study aquaporin physiology (Ben et al., 2008; Zhang J. et al., 2018). Given this, we chose to test the effect of Niclosamide in these cell lines, both to rule out a cell-type specific effect, and because baseline AQP5 levels were higher in these cells. Treatment of A549 cells with Niclosamide for 6 h resulted in increased AQP5 compared to untreated cells (Figures 5C,D). As a complementary approach to investigate our findings in a more relevant human airway model, we utilized primary human bronchial epithelial (HBE) cells grown on an air-liquid interface to test if Niclosamide had a similar effect. Indeed, Niclosamide increased AQP5, but not AQP4, in HBE cells (*n* = 6 wells/condition) (Figures 5E–G). Taken together, these results suggest that Niclosamide stabilizes AQP5 by preventing its ubiquitination and degradation; further, the effects of Niclosamide to increase AQP5 are similarly observed on endogenous protein in A549 and primary HBE cells.

### Niclosamide Prevents AQP5 Loss in Hypotonic Conditions

**FIGURE 6:**
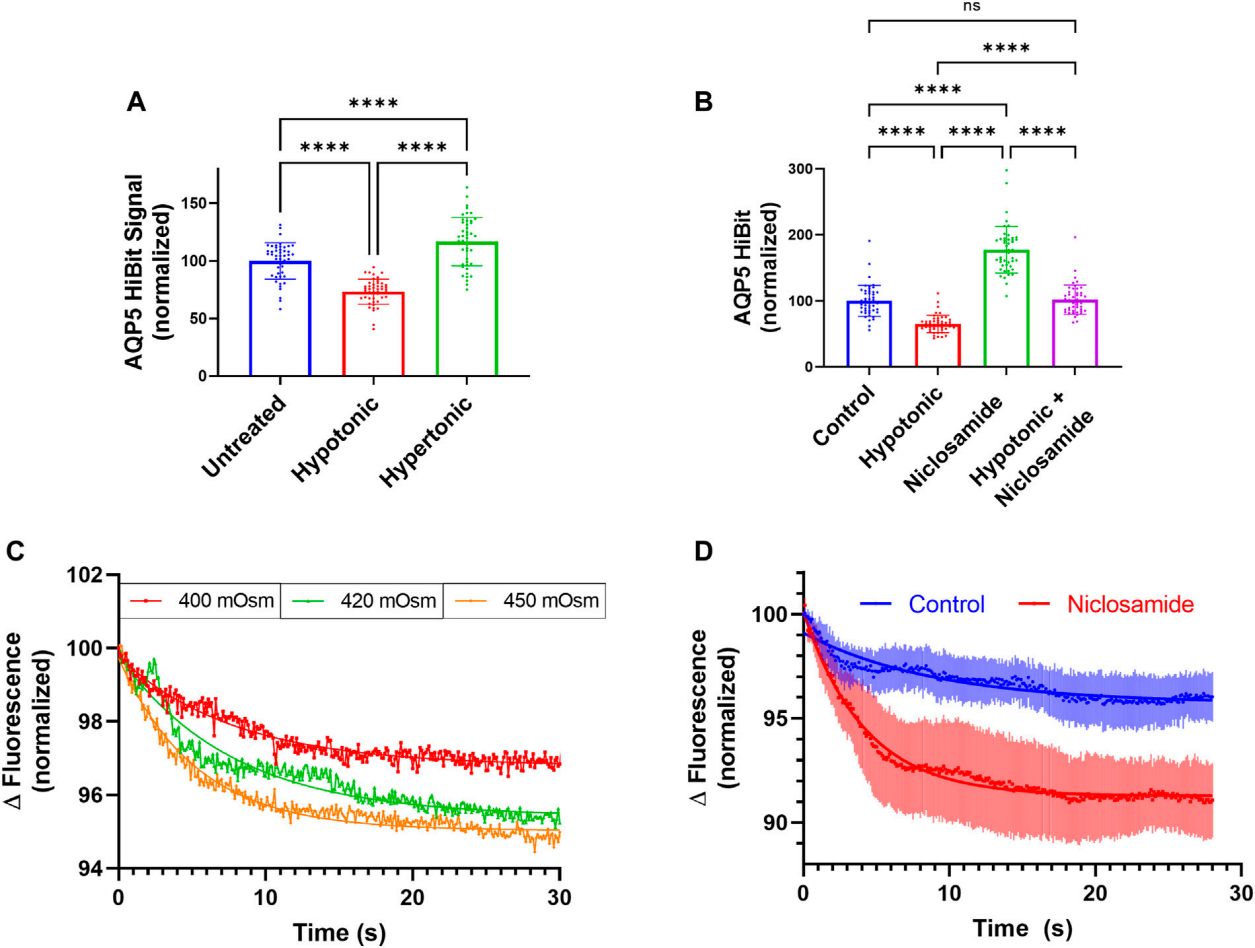
Niclosamide Effect on AQP5 abundance in response to tonicity changes. **(A)** Normalized AQP5-HiBiT luminescence in response to 6-h treatment with hypotonic media (∼150 mOsm), and hypertonic media (∼400 mOsm). N = 48 wells/condition. Graph is representative of repeated independent experiments. **(B)** Normalized AQP5 HiBiT luminescence response to 6-h treatment with hypotonic media, Niclosamide, or combination. N = 48 wells/condition. Graph is representative of repeated independent experiments. *p <0.05, **p <0.01, *** *p* <.001, ****p <0.0001 by 1-way ANOVA with correction for multiple comparisons by Tukey’s method. **(C)** Calcein in A549 cells in response hypertonic stimuli of 400, 420, and 450 mOsm. **(D)** Calcein fluorescence in vehicle or Niclosamide-treated (2 μM, 6 h) A549 cells. Dots represent average values of *n* = 3 wells, with error bars displaying SEM values at a given time-point.

Using AQP5-HiBiT cells, we tested the effect of hypotonic or hypertonic stress on AQP5-HiBiT abundance. Hypotonic and hypertonic stress have opposing effects on AQP5 abundance–hypotonic stress reduces AQP5 (Sidhaye et al., 2006), while hypertonic stress increases AQP5 (Herrlich et al., 2004). These effects occur over the time period of hours. We observed similar effects in AQP5-HiBiT cells, as hypotonic stress reduced AQP5-HiBiT abundance, and hypertonic stress increased AQP5-HiBiT compared to isotonic conditions after 6 h (Figure 6A). Next, we tested whether Niclosamide could prevent hypotonic-induced reductions in AQP5 levels. Indeed, co-treatment with Niclosamide prevented reduction in AQP5-HiBiT levels during hypotonic stress (Figure 6B). Thus, AQP5-HiBiT cells re-capitulate changes in AQP5 levels in response to physiologically relevant stimuli, and the compound Niclosamide ameliorates AQP5 loss in hypotonic conditions.

### Niclosamide Increases Rapid Cell Volume Changes in Hypertonic Media

Aquaporins regulate water transport across cell membranes. The rate and magnitude of cell volume changes immediately following tonicity changes can be estimated by changes in cell volume, and these effects are regulated by aquaporin water channels (Verkman et al., 2014; Madeira et al., 2016; Li and Wang, 2017). Use of the self-quenching fluorescent dye calcein is an established assay to measure cell volume changes (Hamann et al., 2002; Mola et al., 2009). In response to hypertonic media, increased calcein self-quenching and reduced fluorescence is associated with a reduction in cell volume as a result of water transport out of cells. We sought to determine if Niclosamide would affect rapid (0–30 s) cell volume changes. We first subjected calcein-loaded A549 cells to solutions of 400, 420, or 450 mOsm and measured changes in calcein fluorescence over time. Higher tonicities (420 and 450 mOsm) displayed greater cell volume changes after 30 s (representative tracings, Figure 6C). To determine the effect of Niclosamide on this phenomenon, we treated A549 cells in 96 well plates with either vehicle or Niclosamide for 6 h and then assayed cell volume changes after calcein loading in response to hypertonic stimuli at 400 mOsm (Figure 6D). A non-linear regression analysis was performed using 1-phase decay from an average aggregate tracing from *n* = 3 wells/condition. Cells treated with Niclosamide had a more rapid flux of calcein quenching compared to vehicle-treated cells; half-life Niclosamide-treated 4.2 s (95% CI 2.4s–3.6 s) *vs*. vehicle-treated 6.3s (95% CI 4.3 s–10.3 s). Niclosamide-treated cells also displayed a greater absolute change in calcein fluorescence; fluorescence plateau Niclosamide-treated 91.3% (95% CI 90.95%–91.58%) *vs*. vehicle-treated 95.7% (95% CI 95.1–96.1%). Thus, in A549 cells, treatment with Niclosamide resulted in greater AQP5 levels assayed by immunoblot, as well as greater cell volume changes as measured by calcein fluorescence in hypertonic media. These results suggest that increased AQP5 abundance in response to Niclosamide is associated with an increase in functional water transport, providing a link between Niclosamide treatment and the physiologically relevant cellular effect of water transport.

## Discussion

Aquaporins are the principal channels that regulate water transport across membranes. Members of the aquaporin family are expressed in a tissue-specific manner. In the lung, Aquaporins 1, 4, and 5 are predominant (Yadav et al., 2020). While AQP5 knock-out mice have no discernable difference in baseline phenotype, changes in AQP5 have been implicated in the pathogenesis of acute lung injury, as well as in the development of xerostomia, lung submucosal glandular secretions, and in the regulation of sweat (Song and Verkman, 2001; Verkman, 2007; Inoue, 2016; Zhang J. et al., 2018; Hosoi et al., 2020). Additionally, AQP5 expression is regulated by a number of transcriptional and post-translational mechanisms, including inflammatory signals (Towne et al., 2000; Towne et al., 2001; Hasan et al., 2014; Vassiliou et al., 2017), cAMP (Yang et al., 2003), hypoxia, (Kawedia et al., 2013), shear stress (Sidhaye et al., 2008), and tonicity (Sidhaye et al., 2006). Here we use a novel high-throughput screening approach based on AQP5 abundance to identify repurposed drugs that increased AQP5. Thus, these findings could be examined in several models where increasing AQP5 levels would be beneficial, including acute lung injury and xerostomia. In acute lung injury models, AQP5 has been shown to play a role in barrier protection. In a *Pseudomonas Aeruginosa* model of ALI, AQP5 knockout mice had increased lung injury and increased bacterial dissemination compared to WT mice (Zhang et al., 2011a). After tracheal PA instillation, AQP5 KO lungs had increased BAL protein, increased W/D ratios, and increased lung injury scores by histology. Similarly, in a mouse model of LPS instillation, AQP5 levels were reduced compared to control-treated mice. Further, restoration of AQP5 levels with the sedative agent dexmedetomidine, increased lung AQP5 levels and protected against lung injury (Jiang et al., 2015). In addition to its role in acute lung injury, Aquaporin-5 also plays a vital role in submucosal glands. AQP5 deficiency is associated with xerostomia (Satoh et al., 2013), and AQP5 knockout mice have defective saliva secretion (Ma et al., 1999). In the lung, AQP5 similarly regulates submucosal gland fluid secretion, as AQP5 KO mice have reduced submucosal gland fluid secretion, most likely as a result of reduced transepithelial water migration (Song and Verkman, 2001). Thus, modulation of AQP5 could be a therapeutic strategy to treat disorders of glandular secretion, including both hyper-viscous states such as those observed in Cystic Fibrosis, or excessive glandular secretion characteristic of allergic bronchitis (Song and Verkman, 2001).

Aquaporin-5 also has roles outside of water transport in regulating lung physiology, immunology, and in the development of malignancy. In human bronchial epithelial cells, AQP5 was shown to stabilize microtubules (Sidhaye et al., 2012). In models of COPD, AQP5 knock-out mice are protected from cigarette smoke-induced emphysema, in a mechanisms hypothesized to be related to altered epithelial barrier function and reduced neutrophil recruitment to the lung (Aggarwal et al., 2013). Further, AQP5 SNP’s are associated with lung function decline in humans with COPD (Hansel et al., 2010). In the context of lung adenocarcinoma, increased AQP5 assessed by immunohistochemistry in resected tumors was associated with decreased survival (Chae et al., 2008; Zhang et al., 2010). Further, AQP5 over-expression was associated with increased cell migration (Zhang et al., 2010)and mucin production (Zhang et al., 2011b). Thus, outside of regulating water transport, there is great interest in the function of lung aquaporins, including AQP5, in regulating diverse cellular processes.

Our top candidate compound Niclosamide is a pleotropic anti-helminth drug (Schweizer et al., 2018). Much recent work has identified Niclosamide as a potential candidate to treat a number of inflammatory pulmonary diseases, including asthma (Centeio et al., 2021), cystic fibrosis, and COVID19 (Xu et al., 2020; Al-Kuraishy et al., 2021). Interestingly, Niclosamide was recently identified as an inhibitor of the Ca2+ activated Cl-channel TMEM16A which reduced mucus production and secretion, along with causing bronchodilation in murine models of asthma. Thus, through multiple potential mechanisms of action, Niclosamide appears to be an attractive agent to test for effects in several pulmonary inflammatory conditions. Our results suggest that Niclosamide can also affect water transport through stabilizing AQP5 levels. Interestingly, structurally Niclosamide contains a N-phenylbenazmide core, and modified phenylphenzamides have been identified in high-throughput screens as inhibitors of AQP4 function. A modified phenylbenzamide compound (AER-270) inhibited AQP4 function *in vitro* and reduced cerebral edema in models of ischemic stroke. Notably, the authors examined whether AER-270 affected AQP5 function and determined it had nearly no effect on AQP5 function (Farr et al., 2019). Our approach contrasts with this approach in that our screen focused on AQP5 abundance rather than downstream function. Futher, we show that Niclosamide increases AQP5 levels via reduced AQP5 ubiquitination and degradation, increasing total AQP5 levels.

While our study focused on the role of AQP5, several other aquaporin proteins regulate lung physiology, including AQP1, 3, and 4. These aquaporin proteins are expressed in different cell types along the respiratory system and have distince physiological roles in mainataining homeostasis. For example, AQP1 is primarily expressed in endothelial cells lining the respiratory tract and functions to regulate osmotic water transport across capillary membranes, and AQP4 is concentrates in cells in the upper airway (Yadav et al., 2020). While our study does not address *in vivo* changes of other aquaporin channels in response to Niclosamide, our data suggest that Niclosamide does not similarly affect levels of AQP4 *in vitro*. Certainly, the global effect of alveolar fluid clearance in response to Niclosamide or other agents from our screens warrants further investigation.

Our high-throughput screening approach is unique in that it screens for compounds that influence AQP5 abundance. Of note, several studies have shown that increases in functional AQP5 in cell membranes results in increased water transport; thus, an agent targeting AQP5 abundance would most likely result in increased water transport through the channel. This concept is also true in many physiological situations where insertion of aquaporins into the cell membrane from intracellular compartments increases water transport through them. Our study establishes a workflow by which abundance of a desired protein can be measured via high throughput screening. This technique can be more broadly applied to additional proteins to screen for repurposed drugs, novel small molecules, or other stimuli that affect abundance. Here, we validate findings from our screen (performed in stably expressing Beas-2B cells) in another lung epithelial cell line - A549 cells, and in primary human bronchial epithelial cells grown on an air-liquid interface. Further, we show a physiologically relevant change in cell volume in Niclosamide-treated cells. Further studies are warranted to determine if treatment with Niclosamide could alter phenotypes in disease models, such as increasing saliva secretion in models of xerostomia, enhancing pulmonary edema clearance in acute lung injury, and potentially altering the rate of perspiration.

We examined total cellular abundance of AQP5 in immortalized lung epithelial cell lines and in primary, polarized human bronchial epithelial cells. Regulation of aquaporin channels at the cellular membranes is a complex process dependent on several factors. Several aquaporin proteins are regulated via trafficking mechanisms, where an intracellular pool of inactive protein can be rapidly mobilized the cell membrane in response to various stimuli. AQP5 has been shown to be regulated in this fashion, involving cytoskeletal elements in response to intracellular calcium flux (Tada et al., 1999). While our study did not directly examine AQP5 trafficking, and whether Niclosamide affects AQP5 trafficking, results from the calcein cell volume change assay suggest that the increase in total cellular AQP5 results in a change in functional AQP5 at the plasma membrane that increases cell volume changes more rapidly. Thus, whether Niclosamide could also have effects on AQP5 trafficking is an open are of investigation.

Our approach in using a CMV-promoter driven AQP5 construct allowed for identification of compounds that had transcriptionally-independent effects on AQP5 abundance. The top hits of our screen were proteasome inhibitors, and our prior studies have identified agents interfering with protein degradation (Chen et al., 2020a) using this platform. Indeed, we determined that AQP5 ubiquitination was reduced after Niclosamide treatment. While ubiquitination is the mechanism directing protein degradation, the family of ubiquitin ligases (E1, E2, E3 ligases) are responsible for carrying out cellular ubiquitination events. In the case of aquaporins, several different and distinct E3 ligases have been identified to regulate aquaporin ubiquitination. E3 ligases target specific substrates, and other members of the AQP family are targeted by distinct E3 ligases. For example, AQP4 is targeted by the E3 ligase Atrogin-1 (Chung et al., 2020), and AQP1 is targeted by the E3 ligase CHIP (Centrone et al., 2017). The E3 ligases targeting AQP5 are unknown, and it is possible that Niclosamide interferes with the E3 ligase targeting AQP5 for ubiquitination. Further, it is possible that other hits from our screen (Candesartan, Panobinostat) could also affect AQP5 by similar mechanisms. Efforts to identify the AQP5-targeting E3 ligase are ongoing in our laboratory.

In conclusion, this investigation highlights a unique approach to repurposed drug screening by 1) examining protein abundance as a screening endpoint, and 2) designing screens for compound effects on a specific target of interest, i.e. AQP5. Using a library of FDA-approved compounds, we identify that the compound Niclosamide increases AQP5 abundance in lung epithelial cells. We show this effect is likely mediated through a reduction in ubiquitination and proteasomal degradation of AQP5 and demonstrate the drug effect in immortalized A549 cells and primary human bronchial epithelial cells. The findings of this study show how our screening approach based on protein abundance could be applied broadly for nearly any protein target of interest. Our results demonstrating the effect of Niclosamide on AQP5 abundance and cell volume changes in hypertonic media warrant further investigation of this agent in small animal models of disease, including acute lung injury and xerostomia, where increasing AQP5 abundance could be beneficial.

## Data Availability

The raw data supporting the conclusions of this article will be made available by the authors, without undue reservation.
