# Supplementary figures and images for: A Repurposed Drug Screen for Compounds Regulating Aquaporin 5 Stability in Lung Epithelial Cells
Source: Front Pharmacol. 2022 Jan 25;13:828643. doi: 10.3389/fphar.2022.828643 (PMC8821664; doi:10.3389/fphar.2022.828643)

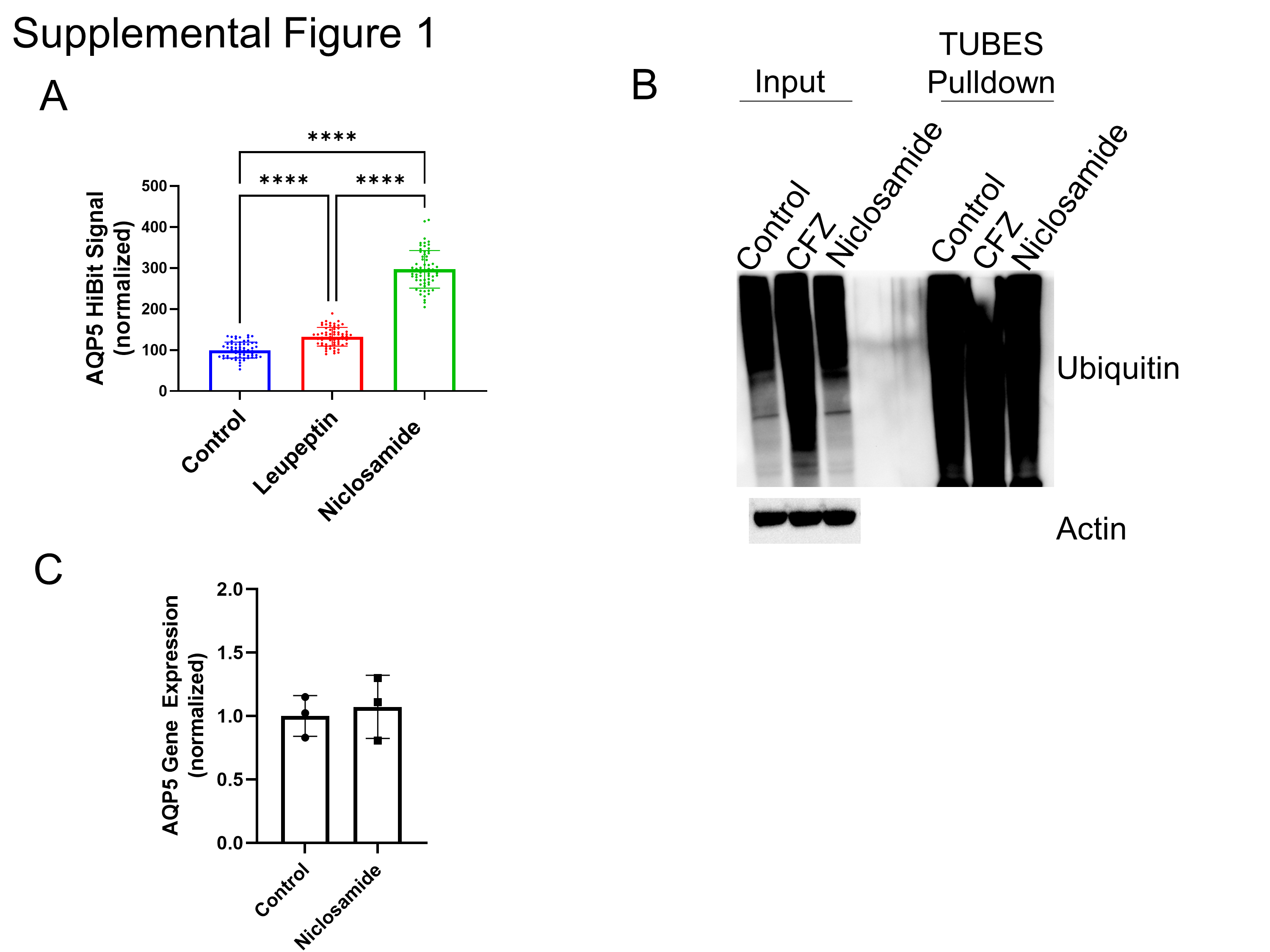

Supplement: Supplementary file 1 [file Image1.TIF]
